# Molecular Dynamics Modeling and Simulation of Diamond Cutting of Cerium

**DOI:** 10.1186/s11671-017-2235-1

**Published:** 2017-07-25

**Authors:** Junjie Zhang, Haibing Zheng, Maobing Shuai, Yao Li, Yang Yang, Tao Sun

**Affiliations:** 1 Center for Precision Engineering, Harbin Institute of Technology, Harbin, 150001 China; 2 Science and Technology on Surface Physics and Chemistry Laboratory, Mianyang, 621908 China

**Keywords:** Cerium, Diamond cutting, Phase transformation, Dislocation, Molecular dynamics simulation

## Abstract

The coupling between structural phase transformations and dislocations induces challenges in understanding the deformation behavior of metallic cerium at the nanoscale. In the present work, we elucidate the underlying mechanism of cerium under ultra-precision diamond cutting by means of molecular dynamics modeling and simulations. The molecular dynamics model of diamond cutting of cerium is established by assigning empirical potentials to describe atomic interactions and evaluating properties of two face-centered cubic cerium phases. Subsequent molecular dynamics simulations reveal that dislocation slip dominates the plastic deformation of cerium under the cutting process. In addition, the analysis based on atomic radial distribution functions demonstrates that there are trivial phase transformations from the γ-Ce to the δ-Ce occurred in both machined surface and formed chip. Following investigations on machining parameter dependence reveal the optimal machining conditions for achieving high quality of machined surface of cerium.

## Background

Cerium (Ce) with an atomic number of 58 is one of the most abundant lanthanide metals. Cerium has wide applications for its intriguing mechanical, physical, and chemical properties. It is known that machined surface morphology of metal parts has a strong influence on their functionality, performance, and life cycle. For instance, the corrosion resistance of metal parts can be effectively improved by reducing surface roughness or introducing compressive residual stress in machined surface [1–3]. More recently, Yan et al. employed a novel tip-based mechanical machining technique to fabricate periodic triangular micro-cavities on Cu(111), which is demonstrated to be a surface-enhanced Raman scattering substrate [4]. Specifically for cerium that is used to store hydrogen [5], the surface finish of cerium strongly influences the reaction between cerium and hydrogen at room temperature. Therefore, achieving high accuracy of machined surface morphology of cerium is crucial for its applications. Ultra-precision diamond cutting is one important manufacturing technique to obtain ultra-smooth surface finish of high surface integrity, ultra-low surface roughness, high flatness, low metallographic structure evolution, and low subsurface damage [6, 7]. However, either experimental or theoretical work about the diamond cutting of cerium had been rarely reported. Furthermore, since in the ultra-precision diamond cutting process, the tool edge radius is comparable with depth of cut, the properties of workpiece material play an important even dominant role in the cutting process. Therefore, the understanding of machining mechanisms of cerium is challenging for its complex deformation behavior.

First, cerium is known for its extraordinary rich pressure-temperature phase diagram driven by the delocalization of 4f electrons. At atmospheric pressure and low temperatures below 110 K, the α-Ce (face-centered cubic (fcc)) is stable. At increased temperatures ranging from 45 to 275 K the α-Ce transforms to the β-Ce (double hexagonal close-packed (dhcp)). The γ-Ce (fcc) is stable at moderate temperatures between 270 and 999 K. At high temperatures between 999 K and the melting temperature of 1071 K the δ-Ce (body-centered cubic (bcc)) is stable [8–11]. In particular, the most fascinating isostructural phase transformation from the trivalent low-density γ-Ce to the much denser α-Ce at 295 K and under 8 kbar is accompanied with a large volume collapse of 20% [8, 12–14]. The phase transformation-induced modification of the electronic structure and bonding configuration in cerium inevitably has a strong impact on its deformation behavior. Specifically, the high temperature and high pressure formed in the contact region between cutting tool and workpiece may result in phase transformation of cerium in the diamond cutting process. Second, metallic cerium has considerable ductility governed by dislocations [15]. It is known that dislocation nucleation and glide play key roles in the plastic deformation of fcc metals under mechanical machining. However, it is still largely unknown about the interaction between phase transformations and dislocations in the diamond cutting of cerium.

The constituents of machining mechanisms consist of microscopic deformation behavior of workpiece material and its correlation with macroscopic machining results in terms of cutting force, chip profile, and machined surface morphology. As an important supplementary to machining experiments, molecular dynamics (MD) simulation has been demonstrated to be a powerful tool for elucidating fundamental mechanical machining mechanisms of different kinds of materials. Li et al. reported that the minimum wear depth of single crystalline Cu(111) under nanoscratching that is equivalent to the critical penetration depth at which plasticity initiates increases with probe radius [16]. More recently, they investigated the mechanical behaviors and deformation mechanisms of AlCrCuFe high-entropy alloys under nanoscratching and reported a larger surface pileup volume than pure metals due to its good high-temperature stability of the alloy material [17]. Gao et al. investigated the generation and evolution of plasticity and defects in orthogonal cutting of a bcc Fe [18]. Zhu et al. reported a size effect on the probe shape dependence of the nanoscratching [19]. Hosseini et al. investigated the effects of tool edge radius on nanomachining of single crystal copper [20]. Liu et al. found that the difference between static and dynamic friction coefficients disappear in single asperity friction of Cu(111) due to the interference between asperities [21]. Romero et al. found that the adhesion during orthogonal cutting of a copper substrate can be reinforced by varying the tool rake angle and by choosing specific lattice orientations [22]. Yang et al. indicated that the abrasive self-rotation velocity and direction have significant influence on the morphology and quality of the machined surface of single crystal copper under polishing [23]. Vargonen et al. reported that the tip height loss per scratching distance during scratching is a function of the normal stress and the tapering angle of the tip [24]. Sun et al. proved impact of GB on the scratching of bi-crystal copper [25]. Chen et al. found that water molecules effectively reduce the friction between the tool and workpiece in the nanometric cutting of copper [26]. Wu et al. reported that the bonding energy has a significant influence on the friction [27]. In addition, as compared to experimental investigations, mechanical properties of each cerium phase can be conveniently studied by means of MD simulations, which is crucial for understanding the interaction between phase transformations and dislocations in cerium. More recently, Zhang et al. investigated the interactions between phase transformation and dislocation at the elastic-plastic transition in silicon nanoindentation by MD simulations [28]. However, to the best of our knowledge, there is no work reported on the MD investigation of mechanical machining of cerium.

Therefore, in the present work, we first establish the MD model of diamond cutting of cerium by constructing atomic configurations of workpiece and tool, assigning empirical potentials for Ce-Ce and Ce-C atomic interactions, and characterizing two fcc phases of cerium. With the established MD model, we then perform MD simulations of diamond cutting of cerium to elucidate the fundamental machining mechanisms of cerium and investigate the influences of rake angle of cutting tool and crystallographic orientation of workpiece on the cutting process.

## Methods

### MD Model of Diamond Cutting

**Fig. 1:**
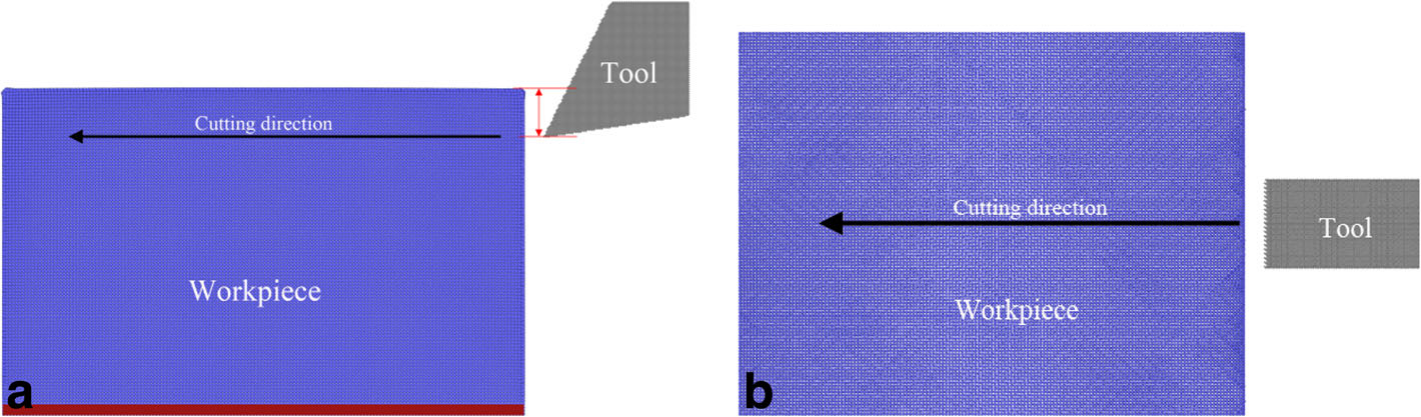
MD model of diamond cutting of cerium; (color online) MD model of diamond cutting of cerium. **a** *Front view* and **b** *top view*. *Red* and *blue colors* stand for bottom and mobile Ce atoms, and *gray color* indicates C atoms

Figure 1 shows the MD model of diamond cutting, which consists of a single crystal cerium workpiece and a diamond cutting tool. The cerium workpiece has a dimension of 41, 25, and 31 nm in horizontal, vertical, and longitudinal direction, respectively, and consists of one million γ-Ce atoms in fcc structure. Periodic boundary condition is only applied in longitudinal direction. The workpiece consists of two types of atoms, as bottom atoms and mobile atoms, respectively. The layer of bottom atoms with a thickness of 2 nm is fixed in space to prevent any rigid motion of the workpiece. The motion of mobile atoms follows Newton equation of motion with velocity-verlet integration algorithm. The temperature of each atom in the workpiece is monitored in the cutting process to represent heat dissipation generated. To address the influence of crystallographic orientation, three cerium workpieces with (010), (110), and (111) free surface in vertical direction are considered. The diamond cutting tool with a sharp edge has a relief angle of 9° and is composed of 0.1 million C atoms in diamond structure. Seven rake angles, as −30°, −20°, −10°, 0°, 10°, 20°, and 30°, are utilized to address the influence of rake angle. Given the ultrahigh hardness of diamond as compared to cerium and ultra-short cutting distance, the wear of diamond tool during the cutting process is not considered. Therefore, the diamond cutting tool is set as a rigid body, i.e., the coordinates and velocities of all the atoms in the cutting tool are updated every time step, in such a way the cutting tool moves as a single entity without any deformation.

There are three types of atomic interactions in the simulated system, as Ce-Ce in the cerium workpiece, Ce-C between the cerium workpiece and the diamond cutting tool, and C-C in the diamond cutting tool, respectively. The C-C interactions are omitted as the diamond cutting tool is treated as rigid body without any deformation in the cutting process. The embedded atom method (EAM) composed of interacting pair potential and electron embedding energy has been widely used to describe metallic systems, which can be expressed as

1 $$ {E}_{\mathrm{tot}}=\frac{1}{2}{\sum}_{i,j}{\phi}_{ij}\left({r}_{ij}\right)+{\sum}_i{F}_i\left({\rho}_i\right) $$

2 $$ {\rho}_i={\sum}_{j\ne i}{\rho}_i\left({r}_{ij}\right) $$

where *r*_*ij*_ is the distance between atoms *i* and *j*, *ϕ*_*ij*_ is the pair potential between atoms *i* and *j*, *F*_*i*_ represents the embedding energy that is generated when an atom *i* is embedded, *ρ*_*i*_ is the electron density at *i* atom generated by all atoms except atom *i*, and *ρ*_j_ is a function of the electron density of atom *j* at atom *i*. The EAM parameters for cerium by Sheng et al. are utilized to describe the Ce-Ce interactions, which is capable of accurately describing bulk elastic properties of fcc cerium phases [29]. The Morse potential is utilized to describe the Ce-C interaction, which can be expressed as

3 $$ {E}_{\mathrm{tot}}={\sum}_{ij}{D}_0\left[{e}^{-2\alpha \left(r-r0\right)}-2{e}^{-\alpha \left(r-r0\right)}\right] $$

where *D*_0_ (0.087 eV) is the cohesive energy, α (5.14) is the elastic modulus, and *r*_*0*_ (2.93 Å) represents the equilibrium distance between atoms *i* and *j*, respectively. The cutoff radius of the Morse potential is chosen as 1.0 nm [30].

The as-created simulation system is first equilibrated to its equilibrium configuration at 30 K and under 0 bar in the NPT ensemble (constant number of atoms *N*, constant pressure *P*, and constant temperature *T*). Then, the equilibrated workpiece is subjected to the diamond cutting with a constant velocity of 100 m/s and a depth of cut of 4 nm in the canonical ensemble (constant number of atoms *N*, constant volume *V*, and constant temperature *T*). The cutting direction is indicated by arrows colored by red in different views point of the cutting model. And the cutting force is defined as the force component along the cutting direction. The utilized depth of cut in the ultra-precision machining experiment is a few micrometers. We note that the simulated dimension of workpiece and depth of cut are several orders of magnitude smaller than that utilized in ultra-precision diamond cutting experiments, due to the limitation of length scale in atomistic simulations. We also note that the employed cutting velocity of 100 m/s in current MD simulations of nanometric cutting is several orders of magnitude higher than typical velocities of tens of micrometers per second utilized in ultra-precision diamond cutting experiments, giving the intrinsic requirement of the integration time step to be of the order of femtosecond (fs). The common neighbor analysis (CNA) is utilized to identify types of lattice defects [31], and the coloring scheme is as follows: green stands for fcc atoms, red for hexagonal close-packed (hcp) atoms, blue for body-centered cubic (bcc) atoms, and gray for other atoms including surface atoms and dislocation cores. All the MD simulations are performed by using the LAMMPS code with an integration time step of 1 fs [32]. And the OVITO is utilized to visualize MD data and generate MD snapshots [33].

### Characterizing of Cerium Phases

In the present work, five cerium phases are considered, as γ, α, β, ε, and δ, respectively. Table 1 lists the structural parameters and relating temperature-pressure conditions for each phase that are collected from literatures [8–11]. Bulk atomic configuration of each phase is then constructed according to Table 1. And then, computational simulations of uniaxial tension, shear, and uniform compression of as-constructed bulk configurations are conducted to derive mechanical properties of different Ce phases. Due to the high stable temperature close to the melting point of cerium, the mechanical properties of the δ-Ce are not calculated as it is difficult to perform mechanical tests on the liquid phase. Table 2 lists the derived elastic constants and mechanical properties of each cerium phase. The calculated Young’s modulus of the single crystalline γ-Ce phase is 24.17 GPa, which is comparable with the experimental value of 36.7 GPa reported in nanoindentation of its polycrystalline counterpart [10]. Furthermore, the calculated values of C44 and 1/2(c11-c12) differ by a factor of 3, which agrees well with the experimental value by using inelastic-neutron-scattering techniques [34]. Table 2 demonstrates that the denser α-Ce has a significantly enhanced mechanical properties as compared to its isostructural low-density γ-Ce phase.

**Table 1:** Structural parameters and stable conditions of cerium phases [8–11]

Phase	Crystal structure	Lattice parameters (Å)	Conditions
γ	Face-centered cubic (fcc)	*a* = 5.1610	*P* = 0, *T* = 270~999 K
α	Face-centered cubic (fcc)	*a* = 4.824	*P* = 0, *T* < 110 K / *P* = 8kbar, *T* = 295 K
β	Double hexagonal close-packed (dhcp)	*a* = 3.6810 *c* = 11.857	*P* = 0, *T* = 45~275 K
δ	Body-centered cubic (bcc)	*a* = 4.11	*P* = 0, *T* = 999~1071 K
ε	Body-centered tetragonal (bct)	*a* = 2.92 *b* = 4.84	*P* = 17.5 GPa, 295 K

**Table 2:** Elastic constants and mechanical properties of cerium phases

Phase	Bulk modulus (GPa)	Young’s modulus (GPa)	Shear modulus (GPa)	C11 (GPa)	C12 (GPa)	C44 (GPa)
γ	23.5	24.17	19.37	31.04	19.32	18.55
α	91.0	90.85	24.92	83.93	74.18	28.94
β	108.24	53.35	2.13	50.76	5.181	18.31
ε	111.73	34.17	15.72	223.27	87.34	11.46

**Fig. 2:**
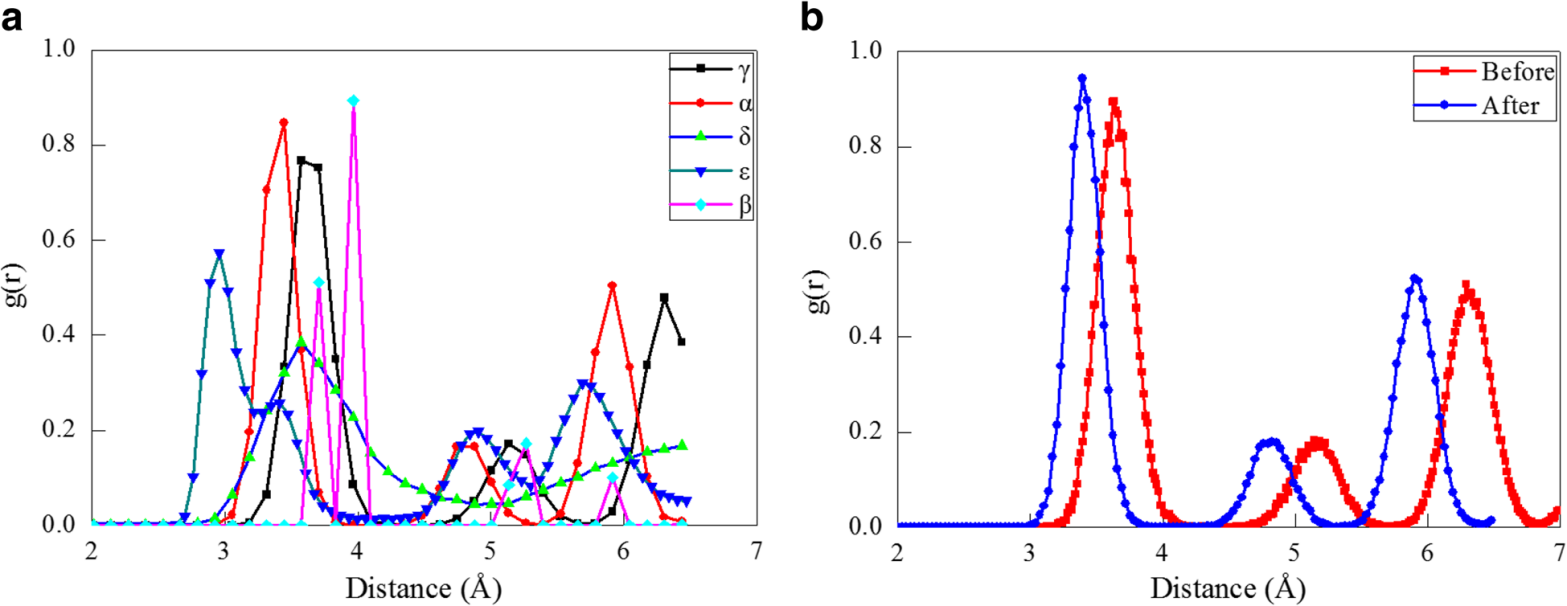
RDF analysis of cerium phases; (color online) **a** RDF of cerium phases. **b** RDF before and after the compression of the γ-Ce

The radial distribution function (RDF), defined as the density variations in a particle system with the distance from a reference particle in the form of sharp peaks. The RDF can be deduced from X-ray spectra and neutron diffraction data [35, 36]. Therefore, it is one of the most important tools for structural characterization of liquids and solids alike and acts as an important link between microscopic atomic arrangements and macroscopic properties. In current study, the RDF is utilized to characterize structural differences between different cerium phases. Figure 2a plots the RDF histograms for the five cerium phases, in which peaks represent characteristics of the corresponding lattice structure: the first, second, and third nearest neighbor distance of the γ-Ce is 3.64, 5.13, and 6.3 Å, respectively; the first, second, and third nearest neighbor distance of the α-Ce is 3.41, 4.85, and 5.92 Å, respectively; the first and second nearest neighbor distance of the δ-Ce is 3.53 and 6.75 Å, respectively; the first, second, third, and fourth nearest neighbor distance of the ε-Ce is 2.96, 3.33, 4.91, and 5.69 Å, respectively; the first, second, third, and fourth nearest neighbor distance of the β-Ce is 3.71, 3.97, 5.27, and 5.92 Å, respectively. To demonstrate the feasibility of predicting phase transformations between two fcc cerium phases by the employed EAM potential parameters, the uniform compression of the bulk γ-Ce until achieving a volume collapse of 20% is performed. Figure 2b presents the RDF before and after the compression, which respectively coincides well with the RDF of the γ-Ce and the α-Ce, indicating the occurrence of the most well-known γ ➔ α phase transformation [12–14].

## Results and Discussion

### Machining Mechanisms of Cerium

**Fig. 3:**
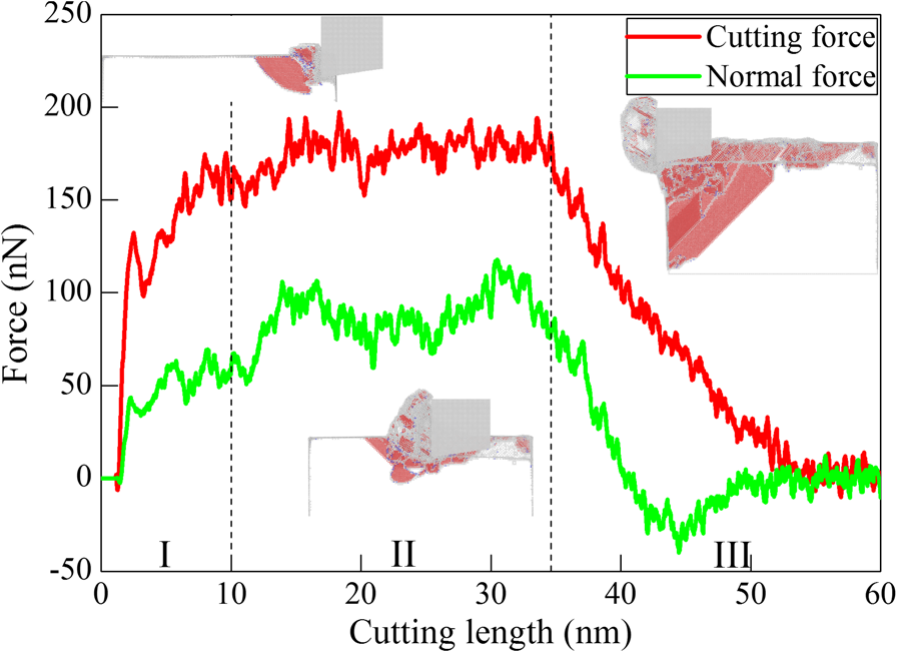
Variation of machining force; (color online) variation of cutting force and normal force with cutting length during the diamond cutting of Ce(010) under a rake angle of 0°. Subfigures present representative defect configurations in different zones, in which atoms are colored according their CNA values

MD simulation of diamond cutting of Ce(010) is first performed to elucidate the fundamental machining mechanisms of cerium. The utilized diamond cutting tool has a rake angle of 0°. There are three components of machining force, as cutting force along horizontal direction, normal force perpendicular to machined surface, and lateral force along longitudinal direction, respectively. Figure 3 shows variations of cutting force and normal force with cutting length during the cutting process, which are categorized into three zones according to cutting length. Accordingly, the subfigure in each zone shows representative cutting configuration, in which atoms are colored according to their CNA values, and fcc atoms are not shown for clear visualization of defects.

It is seen from Fig. 3 that both cutting force and normal force have negative values when the cutting tool is right close to the workpiece, due to the adhesion between cutting tool and workpiece. When the cutting tool begins to contact with the workpiece, the material firstly undergoes elastic deformation, accompanied with rapid increases of both cutting force and normal force. Figure 3 shows that both cutting force and normal force drop rapidly at a cutting length of 2.3 nm, indicating the initiation of plastic deformation. Upon further cutting, both cutting force and normal force increase with strong fluctuations caused by successive nucleation events. It is seen from the subfigure in zone II that there are considerable 1/6<112> Shockley partial dislocation generated in the vicinity of the cutting zone. Both cutting force and normal force mainly fluctuates around constant values in the cutting length ranging from 10 to 35 nm, indicating that the cutting process is stable. And dislocations in zone II mainly reside both in front of and beneath the diamond cutting tool. When the cutting length reaches 35 nm, the cutting tool starts to separate from the workpiece, accompanied with significant decreases of both cutting force and normal force. The subfigure in zone III shows there are considerable dislocations blocked by the left side of the workpiece. Both cutting force and normal force become steady until formed chip is completely separated from the workpiece. Figure 3 shows that during the cutting process, normal force is lower than cutting force.

**Fig. 4:**
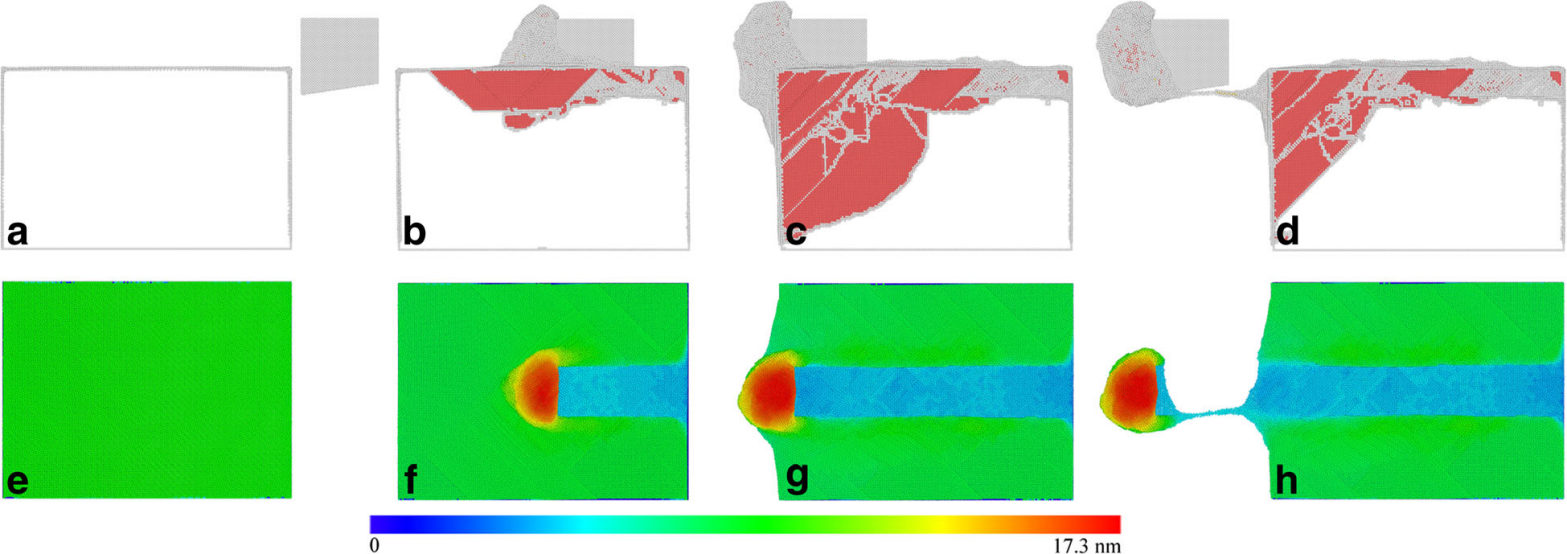
Defect structures and machined surface morphologies; (color online) MD snapshots of instantaneous defect structures (*top row*) and machined surface morphology (*bottom row*) in the diamond cutting of Ce(010) under a rake angle of 0°. Cutting lengths: **a**, **e** 0.0 nm, **b**, **f** 18.8 nm, **c**, **g** 44.8 nm, and **d**, **h** 59.6 nm. Atoms in the *top row* and *bottom row* are colored according to their CNA values and atomic heights, respectively

Figure 4a–d presents instantaneous defect structures within the workpiece at different cutting lengths. Atoms are colored according to their CNA values, and fcc atoms are not shown. Accordingly, Fig. 4e–h presents machined surface morphologies colored by their atomic heights. Dynamic inspection of defect evolution shows that the yielding of workpiece is accompanied by nucleation of 1/6<112> Shockley partial dislocations from the right side free surface and their subsequent glide on adjacent {111} slip planes and along <110> slip directions. The motion of Shockley partial dislocations is accompanied with expansion of stacking faults that are bounded by dislocation cores. With the progress of cutting process, large amount of partial dislocations emits from top free surface in front of the cutting tool, which leads to considerable chips formed along the rake face of cutting tool, as shown in Fig. 4f. Simultaneously, dislocations behind the cutting tool move upwards to annihilate at top free surface, leading to a significant recovery of machined surface. Figure 4c shows that when the cutting tool approaches the left boundary of the workpiece, the propagation of dislocations is strongly blocked by the left side free surface, accompanied with significantly increased chip volume, as shown in Fig. 4g. Figure 4d shows that after the complete separation between chip and workpiece, the dislocation density within the workpiece decreases significantly due to dislocation annihilation at top free surface.

**Fig. 5:**
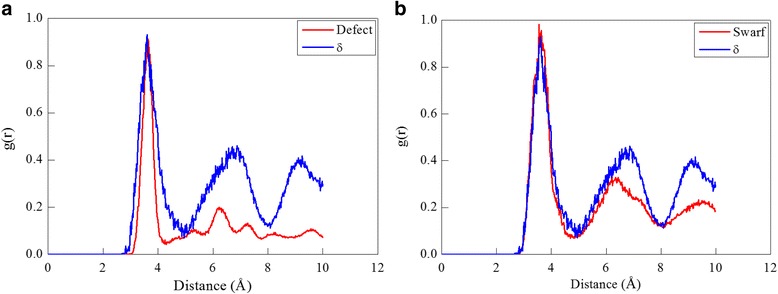
Analysis of phase transformation in cutting process; (color online) analysis of phase transformation in the diamond cutting of Ce(010) under a rake angle of 0°. RDF of **a** workpiece and **b** chip after the cutting

In addition to dislocation slip-dominated plasticity, the probability of phase transformation in the diamond cutting process is also evaluated by performing RDF analysis on defect zone within the workpiece and formed chips. The γ-Ce in fcc structure is first excluded in OVITO by using the CNA algorithm, and the rest atoms are composed of defect zones including bcc, hcp, and other atoms. Then, RDF analysis is performed on the defect zone. And the quantity of different Ce phases can be deduced by number of different types of defect atoms. Figure 5a shows that the three peaks of the RDF of defect zone beneath the machined surface coincide well with the exact three peaks of the RDF of the δ-Ce, suggesting the occurrence of phase transformation from the γ-Ce to the δ-Ce. While the δ-Ce is stable at high temperature and low pressure, the occurred γ ➔ δ phase transformation indicates the high heat dissipation generated in the cutting process. Furthermore, since the δ-Ce has different mechanical properties from the γ-Ce phase, the generated δ-Ce leads to heterogeneous machining in the subsequent cutting process. Figure 5b indicates that there are also δ-Ce atoms detected in the formed chips, primarily due to the high temperature-triggered γ ➔ δ phase transformation in the contact region between the formed chip and rake surface of the cutting tool. However, the quantity of formed δ-Ce in both the defect zone and chip is very trivial, indicating that phase transformation is not prominent in the diamond cutting of cerium.

### Influence of Rake Angle

**Fig. 6:**
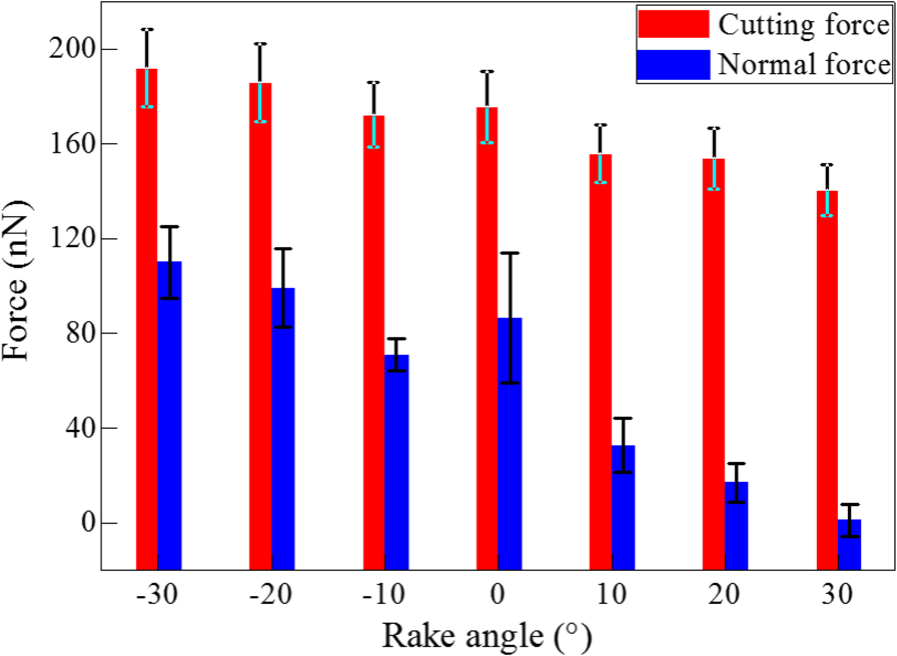
Rake angle dependence of machining force; (color online) influence of rake angle on machining force

With the fundamental understanding of machining mechanisms of cerium, the influence of rake angle of cutting tool on the diamond cutting is studied. Figure 6 plots averaged values of both cutting force and normal force for the seven rake angles. The averaged value of each force component is calculated by averaging instantaneous force values in the cutting length ranging from 10 to 35 nm. Figure 6 shows that the cutting force is higher than the normal force for each rake angle. However, the differential value of the two force components is more pronounced for larger rake angle. Furthermore, both cutting force and normal force decrease with increasing rake angle. According to the Merchant’s theory, with the increase of rake angle, the shear plane angle corresponding the minimum energy also increases, which accordingly lowers the cutting force [37]. The rake angle-dependent machining force variation revealed by current MD simulations agrees well with the Merchant’s theory.

**Fig. 7:**
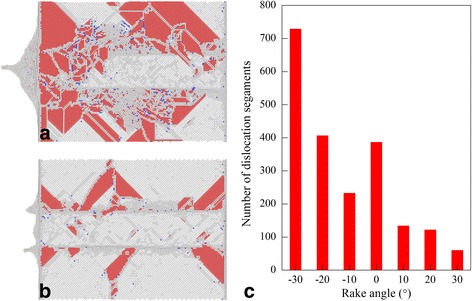
Defect structures generated with different rake angles; (color online) MD snapshots of defect structures after cutting of Cerium with rake angle of **a** −30° and **b** 30°. Atoms are colored according to their CNA values. **c** Rake angle-dependent dislocation number

Figure 7a, b presents bottom view of defect structure beneath the machined surface after completion of diamond cutting process with a rake angle of −30° and 30°, respectively. For each rake angle, both dislocation types and the geometry of dislocation with respect to free surface are the same. However, the dislocation density is significantly higher for the rake angle of −30° than the rake angle of 30°, indicating a more complex plastic deformation occurred. Figure 7c presents the number of dislocation segments remained within workpiece after diamond cutting with different rake angles, indicating that the dislocation density decreases with increasing rake angle. It should be noted that different types of dislocations categorized by the Burgers vectors, including 1/2<110> perfect dislocation, 1/6<112> Shockley partial dislocation, 1/6<110> Stair-rod dislocation, and 1/3<111> Frank partial dislocation, are taken into consideration in Fig. 7c. In nanometric cutting process, the dislocation-mediated microscopic deformation of workpiece material has a strong correlation with macroscopic machining results in terms of machining force and machined surface morphology. For instance, interaction and reaction of dislocations leads to formation of sessile dislocation structures that block subsequent dislocation motion; consequently, the resulting work hardening leads to increase of machining force. The annihilation of dislocations at free surface leads to recovery of machined surface, accompanied with formation of surface pile up [38].

**Fig. 8:**
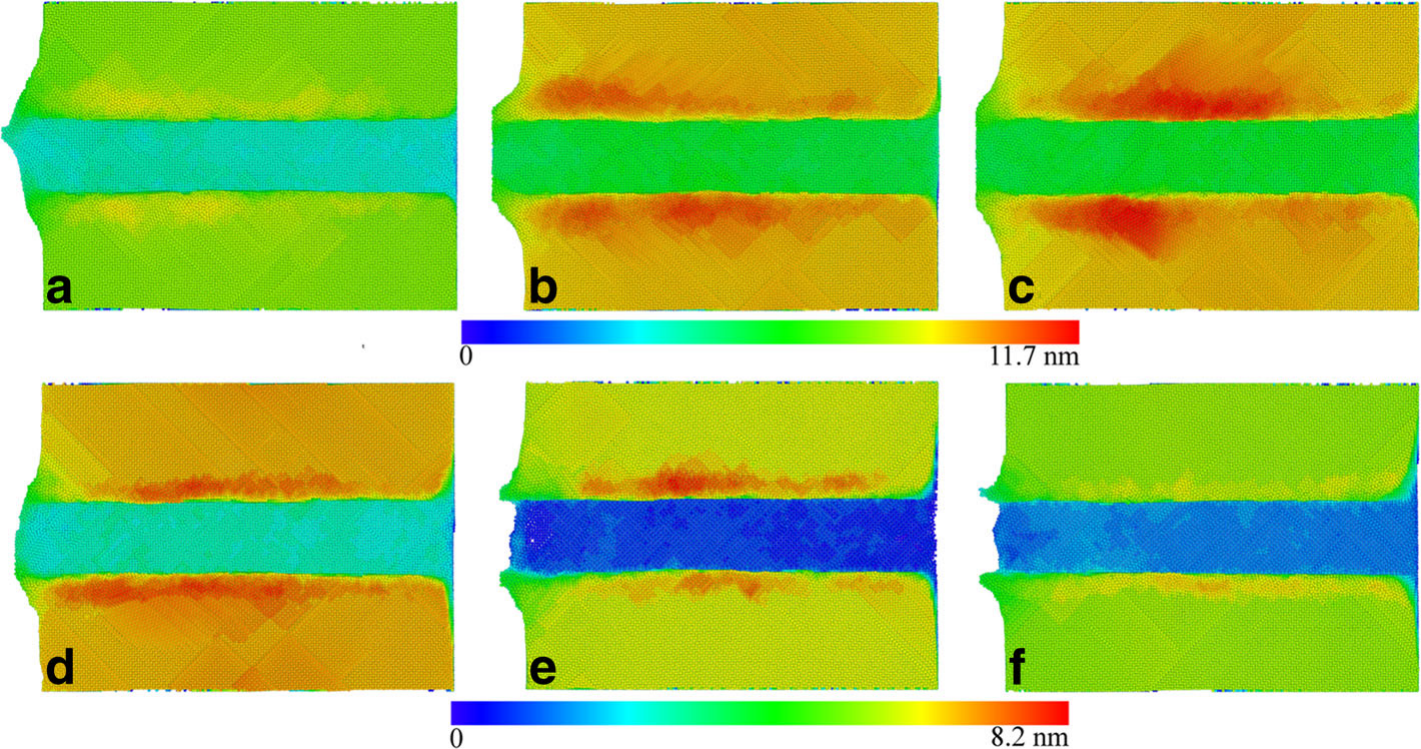
Rake angle dependence of machined surface morphologies; (color online) machined surface morphology with different rake angles: **a** −10°, **b** −20°, **c** −30°, **d** 10°, **e** 20°, and **f** 30°. Atoms are colored according to their atomic heights

Figure 8 presents machined surface morphologies after the completion of cutting processes for different rake angles. The volume of surface pile up on both sides of formed groove is more pronounced for the rake angle with negative values than that with positive ones. Furthermore, the distribution of surface pile up is less uniform for negative rake angle than for positive rake angle. Figure 8c clearly shows the asymmetry distribution of surface pile up along the formed groove for the rake angle of −30°. It is seen from Fig. 8 that the volume of surface pile up decreases with increasing rake angle. Therefore, it is indicated that the rake angle of 30° is optimal for the diamond cutting of cerium for the lowest machining force, the lowest dislocation density, and the lowest surface pile up than the other rake angles.

### Influence of Crystal Orientation of Cerium Workpiece

**Fig. 9:**
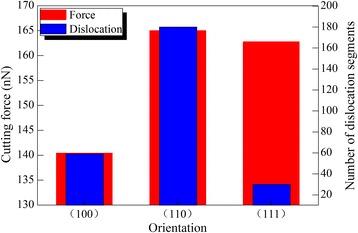
Crystal orientation dependence of cutting force and dislocation number; (color online) crystal orientation dependence of cutting force and dislocation number

The influence of crystal orientation on the diamond cutting of cerium under the optimal rake angle of 30° is also investigated. All the machining parameters are the same for Ce(010), Ce(110), and Ce(111). Figure 9 shows variations of cutting force and dislocation number with different crystal orientations. The cutting force of Ce(010) is significantly lower than that of Ce(110) and Ce(111). Although the dislocation number is the lowest in Ce(111), the dislocation density in Ce(010) is significantly lower than that in Ce(110). It is known that the geometry between slip plane and free surface varies with crystal orientation. For both Ce(010) and Ce(110), the four {111} slip planes are inclined to machined surface. However, there is one {111} slip plane parallel to machined surface of Ce(111), in addition to three {111} inclined slip planes. While microscopic deformation of workpiece material is dominated by dislocation slips, the observed macroscopic machining results in terms of machined surface and machining force can also be influenced by machined surface morphology. Although the easy glide of dislocations on the {111} slip plane parallel to free surface is energetically favorable for the accommodation of plastic strain caused by cutting tool action, the resulting considerable surface pile up increases the machining resistance, which leads to a high cutting force.

**Fig. 10:**
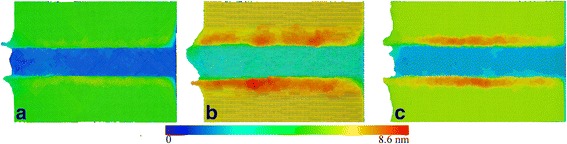
Crystal orientation dependence of machined surface morphology; (color online) crystal orientation dependence of machined surface morphology. Atoms are colored according to atomic heights. Crystal orientation. **a** (010). **b** (110). **c** (111)

Figure 10a–c presents machined surface morphology of Ce(010), Ce(110), and Ce(111), respectively, indicating that the crystal orientation has a strong influence on machined surface quality. The volume of surface pile up is the smallest for Ce(010), followed by Ce(111), and the largest for Ce(110). Correspondingly, the material removal in the form of chip is the most pronounced for Ce(010). Furthermore, it is seen that the surface pile up of Ce(111) on both side of formed groove presents the highest symmetry, while that of Ce(110) is the worst. Therefore, it is indicated that the crystal orientation of (010) is optimal for the diamond cutting of cerium due to its low machining force, low dislocation density, and low surface pile up.

## Conclusions

In summary, we perform MD modeling and simulation to elucidate the underlying mechanisms of cerium under the ultra-precision diamond cutting. The EAM and Morse potentials are respectively employed to describe atomic interactions within cerium workpiece and the interactions between cerium workpiece and diamond cutting tool. The elastic constants, mechanical properties, and propensity of phase transformation of cerium phases are evaluated, which demonstrates the feasibility of predicting phase transformation of cerium by the current established MD model. Subsequent MD simulations of diamond cutting reveal that the plastic deformation of cerium is governed by dislocation nucleation and subsequent glide, which is similar with other fcc metals. In addition, there is γ ➔ δ phase transformation occurred within both machined surface and formed chip. It is found that high quality of machined surface and low machining force can be achieved in the diamond cutting of cerium with the optimal machining conditions, i.e., a rake angle of 30° for a crystal orientation of (010).
